# Removal of CO_2_ from Biogas during Mineral Carbonation with Waste Materials

**DOI:** 10.3390/ijerph20095687

**Published:** 2023-04-28

**Authors:** Paulina Rusanowska, Marcin Zieliński, Marcin Dębowski

**Affiliations:** Department of Environmental Engineering, Faculty of Geoengineering, University of Warmia and Mazury in Olsztyn, 10-720 Olsztyn, Poland; marcin.zielinski@uwm.edu.pl (M.Z.); marcin.debowski@uwm.edu.pl (M.D.)

**Keywords:** methane enrichment, mineral carbonation, calcium oxide

## Abstract

Biogas represents a source of renewable energy that could provide a replacement for fossil fuels to meet the increasing demand for energy. The upgrading of biogas through the removal of CO_2_ to a content of 95–97% of CH_4_ is necessary to increase its calorific value. This review focuses on biogas upgrading technologies using wastes or residues that enable the performing of mineral carbonation. In this research, we analyzed a natural biogas or synthetic one with a content of about (40–50%) of carbon dioxide. The chemical absorption is also briefly described in this study, due to its being the first step in innovative absorption and regeneration processes using mineral carbonization. Wastes with high calcium contents, i.e., ashes, steel-making slags, and stabilized wastewater anaerobic sludge, were considered for direct carbonization, taking into account the leaching of particles from carbonated wastes/residues. Moreover, the different types of reactors used for mineral carbonation have been described. The presented technological solutions are easy to use and economical, and some of them also take into account the regeneration of reagents. However, in the context of their direct use in biogas plants, it is necessary to consider the availability of wastes and residues.

## 1. Introduction

The limited availability and, above all, the rising prices of fossil fuels have become the driving force behind the worldwide development of government policies, prompting research into alternative sources of renewable energy. The composition of biogas that is obtained during anaerobic digestion depends on the types of substrates used and the conditions in the digester [1]. The typical composition of biogas includes CH_4_ (50–70%), CO_2_ (30–40%), H_2_ (5–10%), N_2_ (1–2%), H_2_O (0.3%), H_2_S (0–10.000 ppmv), NH_3_ (0–100 ppmv), hydrocarbons (0–200 mg/m^3^), and siloxanes (0–41 mg/m^3^) [2,3,4]. The high calorific value of CH_4_ (37.78 MJ/m^3^) means that biogas is a good renewable energy source [5]. The calorific efficiency of biogas depends on its CH_4_ concentration, which can be improved by removing CO_2_ from the biogas. Upgraded biogas that contains 95–97% of CH_4_ and 1–3% of CO_2_ is called biomethane. The larger contents of CO_2_ in biogas increases the cost of compression and transportation and might also cause difficulties by freezing at the flow control valves and metering points. The obtained biomethane can be used for heating purposes, for electricity generation, and as fuel for use in vehicles or engines [6].

There are four different types of technology available for upgrading, including absorption (water or amine scrubbing), adsorption (pressure swing adsorption), membrane separation, and cryogenic separation [7,8]. The current commercial technologies for biogas enrichment increase the biogas production costs by 20–72%, due to energy demand and chemical and water requirements, and can cause up to 8% of methane loss [9]. Therefore, due to the high costs for pumps, control and safety equipment, and chemical analyses, using these technologies in small-scale biomethane plants of less than 1000 Nm^3^/day is not economically viable [10].

Mineral carbonation is the strategy for carbon capture and storage (CCS) that has been most used and studied worldwide for over 20 years. During this process, CO_2_ reacts with calcium or magnesium oxide over several reactions and results in solid carbonate formation [11].

$$\mathrm{CaO}+H_{2}O\;\to \mathrm{Ca}{\left(\mathrm{OH}\right)}_{2}\;\mathrm{Ca}{\left(\mathrm{OH}\right)}_{2}\;\to {\;\mathrm{Ca}}^{2+}\;2{\mathrm{OH}}^{-}\;{\mathrm{CO}}_{2}+{\;H}_{2}O\;↔\;2H_{2}{\mathrm{CO}}_{3}\;↔\;2H^{+}+{\;\mathrm{HCO}}_{3}^{-}\;{\mathrm{HCO}}_{3}^{-}+{\;\mathrm{OH}}^{-}\;↔{\;\mathrm{CO}}_{3}^{2-}+{\;H}_{2}O\;{\mathrm{Ca}}^{2+}+{\;\mathrm{CO}}_{3}^{2-}\;\to {\;\mathrm{CaCO}}_{3}$$

Possible sources of calcium for CO_2_ removal are industrial solid wastes (e.g., fly ash, combustion residues, and steel-making slag). These wastes are characterized by alkalinity, which facilitates the process; moreover, they are widely available in industrial areas [12,13]. Summarizing the advantages of the carbonation of CO_2_ are the high stability of the main reaction product containing CO_2_ (CaCO_3_), high cost efficiency, and the possibility of using wastes/residues as a source of calcium or magnesium. The utilization of CaCO_3_ depends on its physicochemical characteristics, such as polymorphism, morphological structure, particle size, etc. The most well-designed CaCO_3_ products could help reduce the total cost of the CO_2_ mineralization process [14]. The newest and most innovative technologies utilizing wastes or residues for the removal of CO_2_ or for the regeneration of reagents during the removal of CO_2_ are presented in this review.

The aim of this review is to present possibilities for using wastes/residues for CO_2_ removal from biogas to achieve biomethane standards during mineral carbonation.

## 2. Materials and Methods

This systematic literature review is intended to summarize the current data regarding CO_2_ removal from biogas in the process of mineral carbonation. The criterion for searching for the desired articles was the accuracy of the search. The selected keywords for the Scopus search were “CO_2_ removal” and “carbonation”. Among the obtained search results, the articles that described the results of experiments where pure CO_2_ was used were excluded. The focus of this review was on biogas with a CH_4_ content of 50–70% and a CO_2_ content of 30–50%; both synthetic and natural forms were considered. The second exclusion was of articles concerning the utilization of waste/residue materials. Many articles described experiments related to the use of minerals as a source of calcium, which was not the subject of this review; therefore, these data were not taken into account. The Scopus search of the keywords “CO_2_ removal” and “carbonation” provided 489 results. After taking into account the above considerations, the 72 most relevant results were chosen; the following article has been written based on these results.

## 3. Chemical Absorption

Biogas containing a mixture of CO_2_ and CH_4_ can be subjected to a process of mineral carbonation during chemical absorption. In the reactor, where the absorption is taking place, the gas is transferred to the gas–liquid interface and is then transferred to the liquid phase. The reagents used during this process include alkaline and alkanolamine. In the aqueous solution, the dissolved CO_2_ reacts with reagents following a reaction mechanism:

$${\mathrm{CO}}_{2}+2{\;\mathrm{OH}}^{-}\to {\;\mathrm{CO}}_{3}^{2-}+{\;H}_{2}O\;{\mathrm{CO}}_{2}+{\;\mathrm{CO}}_{3}^{2-}+{\;H}_{2}O\;\to 2{\mathrm{HCO}}^{3-}\;{\mathrm{CO}}_{2}+{\;R\text{-}\mathrm{NH}}_{2}+{\;H}_{2}O\;\to {\;R\text{-}\mathrm{NH}}_{3}^{+}+{\;\mathrm{HCO}}_{3}^{-}\;{\mathrm{CO}}_{2}+R\;R^{\prime }\text{-}\mathrm{NH}\to {\;\mathrm{RR}\text{-}\mathrm{NCOO}}^{-}+{\;H}^{+}.$$

The efficiency of these reactions depends strongly on the pH, the CO_2_ concentration, and other factors. Several reagents, such as sodium hydroxide (NaOH), calcium hydroxide (Ca(OH)_2_, mono-ethanolamine (MEA), and solid calcium oxide (CaO) were effective in the removal of CO_2_ from biogas. However, the absorption capability declined rapidly with time. The saturation of Ca(OH)_2_ was achieved in 50 min, which was faster than that for NaOH and MEA. The loading for CO_2_ was from 0.18 to 0.22 kg/kg of chemicals [15,16]. In another study, biogas was introduced from a chamber with Ca(OH)_2_ to a chamber with NaOH and was then introduced to a chamber with KOH; at the end of this process, a silica gel bed was used for collecting the water vapor [17]. The biogas composition determined the biogas flow rate and the concentrations of the solutions used for upgrading. The highest CH_4_ content (94.80%) was obtained when the biogas flow rate was at a minimum (0.54 Nm^3^/h) and the solvent concentration was at a maximum (1 N). Calcium hydroxide seems to be the best solution for a biogas upgrading plant. However, this material has a major issue that inhibits its use on a larger scale: the complexity of the regenerative process. This problem makes it impossible to use a regenerative technology for an upgrading plant; this means that the exhausted calcium hydroxide must be replaced with new calcium hydroxide after the adsorption process, in order to make the plant work continuously [18]. Therefore, researchers are looking for cost-saving solutions. One of the proposed methods uses untreated groundwater to prepare the absorbent material [19]. In the pilot-scale study, the removal efficiency of CO_2_ was not significantly different when using groundwater and softened water to prepare NaOH, even if 0.3–0.4% of the NaOH precipitated in the groundwater solution. A concentration of NaOH at 4 g/L was required for the 99% removal of CO_2_. Cost savings could be also ensured by using well-designed CaCO_3_ products, the utilization of which depends on their physicochemical characteristics, such as polymorphism, morphological structure, and particle size [14]. The conditions of mineral carbonation with amines determines the characteristics of the CaCO_3_ products. The mechanisms of amine-promoted carbonation are enhanced by the higher concentrations of MEA and Ca(OH)_2_, which also influences crystal sizes and shapes. Moreover, the amine type influenced the CO_2_ removal efficiency and CaCO_3_ purity. The primary and secondary amines presented a higher CO_2_ absorption kinetic. MEA improved the carbonation process via Ca(OH)_2_ dissolution and calcium leaching. The most efficient conditions for high CaCO_3_ purity (87%), CO_2_ removal efficiency (98%), diverse crystals, and an amine loss of nearly zero in the CaCO_3_ products were achieved with 0.5 M piperazine, 0.1 M Ca(OH)_2_, and a temperature of 55 °C [14].

Chemical absorption can easily be used for biogas upgrading; however, it is not economical, and there is an environmental threat when regeneration of the reagent is not possible.

## 4. Indirect Mineral Carbonation

The following chemicals, comprising MEA, NH_4_OH, CaO, Ca(OH)_2_, KOH, NaOH, FeCl_2_, FeCl_3_, FeSO_4_, Fe_2_O_3_, Fe(OH)_3_, and ZnO, can be used for CO_2_ absorption [20]. However, it is necessary to consider the regeneration methods of these reagents to protect the environment from additional waste. The methods used for regeneration include thermal decomposition, ion exchange operations using anionic resin, and electrodialysis [20,21,22,23]. However, alternative methods employing chemical regeneration are currently being proposed [24,25,26,27]. Briefly, the CO_2_ is removed from the biogas via chemical absorption with NaOH or a KOH solution, to form sodium carbonate (Na_2_CO_3_) or potassium carbonate (K_2_CO_3_). Next, the obtained solution of Na_2_CO_3_ or K_2_CO_3_ is introduced into a precipitation reactor, where the precipitant agents containing Ca(OH)_2_ are added. 

In the regeneration step, to reduce the cost of the process, industrial residues with high calcium content, such as steel slags [24,28] or air pollution control (APC) residues from waste incineration [26,29], are used. These industrial residues and wastes are classified as hazardous wastes; however, carbonated residues should reach non-hazardous status. This process is effective in terms of CO_2_ removal from biogas and storing it in a solid and stable phase (calcite). Similar removal efficiencies were obtained for both reagents (KOH or NaOH) and the regenerated solutions, which could significantly reduce the use of raw chemicals. The regeneration process consists of three stages: washing the pre-treatment residues/wastes, the regeneration reaction, and the final washing of the solid product. Each stage should also be followed by a separation of the liquid and solid, performed by vacuum filtration. The overall regeneration efficiency proved to be limited to 60%. The reason for this low efficiency could be the dilution effect, which can be improved by drying the washed residues or by increasing the recovery of the regenerated solution. However, it transpired that saving the cost of using waste materials generated other costs connected with the washing treatments that appeared to be necessary when using industrial residues. Reusing the post-washing wastewater for the pre-washing process could help reduce the amount of water used. The calculated specific upgrading cost with APC residues is too high when compared with the specific cost of conventional technologies [30]. The main reason for this is that the additional costs for wastewater treatment need to be taken into consideration. 

A high content of calcium enables better precipitation–regeneration efficiencies, whereas a high content of magnesium seems to be less effective [31]. Calcium chloride was proposed as an efficient alternative precipitating agent [32,33]. The source of the CaCl_2_ could be brine [34,35] or residual CaCl_2_ solutions from potassium chlorate (KClO_3_) production [36], or distiller waste from the ammonia–soda process [37]. Precipitation efficiencies of between 62 and 93% were obtained when using CaCl_2_. The conditions that ensured the best balance precipitation efficiency were a molar ratio of CaCl_2_/Na_2_CO_3_ of around 1.2, a time of 30 min, and a temperature of 50 °C [33].

## 5. Direct Mineral Carbonation

Direct mineral carbonation is the most straightforward process route for gas–solid carbonation, which requires the introduction of high-pressure CO_2_ for upgrading the system. Therefore, the aqueous pretreatment of solids is usually necessary [11].

### 5.1. Ashes

Incineration is widely used for the treatment of municipal solid waste. In this thermo-chemical process, energy is recovered from wastes. However, the process generates a huge amount of ash, which is divided into bottom ash and fly ash. Bottom ash is the major and relatively less toxic fraction [38] due to its containing lower heavy-metal concentrations and due to leaching. The ashes contain approximately 22–53% of CaO and consist of small particles, which makes them a good source of calcium for use in carbonation [11,39,40,41]. The using of ashes for the removal of CO_2_ is based on the principle that when in the presence of moisture, calcium oxide reacts with the CO_2_ from the flowing gas to form solid calcium carbonate (CaCO_3_). To achieve this moisture, the ashes are mixed with water before being used for mineral carbonation. The ratio of water to ash (L/S) for slurry-making is about 10:1 [42]. The carbonation potential of slurry bottom ash was found to be dependent on the Ca/Si content: the lower Ca/Si content will result in lower carbonation potential [43]. The optimal conditions for mineral carbonation using ashes are a temperature of 25 °C for 8 h, which results in the CO_2_ uptake of 23.5 mL CO_2_/g by the bottom ash (Table 1) [42].

The ash from a boiler fed with palm oil mill solid residue was also used for CO_2_ removal [44]. The authors presented a zero waste solution where oil palm ash (OPA) was used to enhance CO_2_ removal from biogas by scrubbing with maturation pond effluent (MPE) and, furthermore, the treatment of biogas scrubber effluent (BSE) via *Ceratophyllum demersum* L. (hornwort) cultivation. Oil palm ash contains about 9.65% of CaO [45]. The ratio of MPE (liquid) to OPA (solid) was 0.7:1 kg for making the slurry. The obtained slurry was characterized by high alkalinity, which was favorable for efficient mineral carbonation. The slurry was about 60% more efficient at CO_2_ removal than MPE. The reduction of the CO_2_ in the biogas was 53% when using mixed slurry (Table 1). The biogas flow rate was 300 L/h and the most efficient tested slurry flow rate was 210 L/h [44].

Among the ash from the incineration processes of coupled kilns, conventional kilns, and wood ash, the most suitable ash for biogas upgrading is wood ash [46]. Ash obtained after the incineration of wood and municipal solid waste consists of small particles (d50 < 0.2 mm) and is characterized by a higher porosity than other ashes [46,47]. Wood ash is characterized by a high CaO content (24–46%) [11]. The specific CO_2_ uptake achieved with wood ash is an order of magnitude higher compared to bottom ash [48,49]. This could be related to the physicochemical properties of this ash, including a high content of phases that are reactive with CO_2_. Mineral carbonation with wood ash is able to completely remove CO_2_ from biogas for about 30 h at an inlet gas volumetric flow rate of 24 NL/h and CO_2_ concentration in the biogas of 41–45%. After 30 h, the CO_2_ started to appear again in the outlet stream and its concentration rapidly increased. The specific CO_2_ uptake was about 200 g/kg of dry wood ash (Table 1) [47,50]. In another study, CO_2_ started to appear in the outlet stream after 50 h with an inlet gas volumetric flow rate of 280 NL/h and a CO_2_ concentration in the biogas of 38%. However, after 95 h of upgrading, the limits of the biomethane composition were still maintained for both the total sulfur trace compounds and CO_2_. The specific CO_2_ uptake was 115 g CO_2_/kg of ash (Table 1) [48]. Both experiments used wood ash with a moisture content of 20%. The dry absorption process required a higher mass of activated wood ash, which was increased from 2.5 to 35 g and led to an increase in the removal of CO_2_ from 8.9 to 67.9% [51]. The specific requirement of wood ash per unit of volume of processed gas was about 7 kg/Nm^3^. Annually speaking, for CO_2_ removal from 100 Nm^3^/h of biogas, about 5500 tons of wood ash would be necessary [47]. Therefore, such an upgrading unit using wood ash should be favorable for small-scale plants.

Instead of water being used for slurry preparation, a potassium glycinate solution was proposed by the authors of [13]. The utilization of potassium glycinate solution increased the CO_2_ uptake, Ca^2+/^Mg^2+^ leaching, and CaCO_3_ formation. The high-specificity CO_2_ removal of 275 g/kg coal fly ash required a temperature of 55 °C and 100 g of ash for 1 L of 0.5 M glycinate [13].

The products of mineral carbonization are characterized by reduced toxicity and leaching, which results from the reduced alkalinity and extraction of heavy metals from the ashes in water after the carbonation reactions. The carbonation of the fly ash decreased the leaching of Pb, Cu, Zn, and As, but increased the leaching of Cd and Sb. These results can be used for the determination of optimum pH for a carbonation value of 9.5–10.5. The release of soluble sulfates, chlorides, and fluorides changed little following carbonation [52]. The carbonated bottom ash significantly reduced the leaching of heavy metals; therefore, its eco-toxicity was lower compared with raw bottom ash [42]. The pH of the carbonated wood ash was also reduced by 2 or 3 units [50]. The leaching of Ba was lower over two orders of magnitude. However, the leaching of Cr was slightly affected, while the leaching of V increased [47]. The carbonated wood ash might be applicable for use as a fertilizer; however, this should originate from the combustion of untreated wood [53]. The phototoxicity of the carbonated ashes depends on their controlled dosage. The dosage of the carbonated biomass ash should not exceed 100 g/L; however, the dosage of the carbonated coal fly ash should not exceed 10 g/L [13].

### 5.2. Steel-Making Slag

Steel-making processes generate significant amounts of CO_2_ (1.85 tons of CO_2_ per ton of steel), accounting for 8% of the global CO_2_ emissions in 2020, as presented in public reports published by the World Steel Association. Slags form as a result of interactions between process impurities (primarily silica) and lime at various stages of steel production. The main types of slags produced in the steelmaking process are basic oxygen furnace slag (BOF) (62% of total steel slags), electric arc furnace slag (EAF) (29%), and ladle slag (LS) (9%). The mineralogical composition and solubility characteristics of slags represent very distinct leaching behaviors, including differences in: (i) the amount of heat generated during their dissolution, (ii) their buffering capacity, (iii) the rate and extent of calcium and magnesium extraction from the slags, and (iv) the mineralogical composition of the non-dissolved residues. These findings suggest that separate leaching processes may need to be developed for the different types of slags [54]. Steel-making slag is a potential alkaline adsorbent for the removal of CO_2_ from biogas, due to the presence of free basic oxides such as CaO (about 15–42%) and MgO (5–11%) in its chemical composition [12,55,56,57]. Steel-making slag, despite the above characteristic, is a more challenging source of calcium. Only 5% of the calcium from the steel-making slag was released to the alkaline slurry, which was obtained from 400 g of steel-making slag, mixed with 1 L of water. Based on this finding, 1 ton of steel-making slag would be necessary for the upgrading of 10 m^3^ of biogas to over 90% of methane content [58]. Optimized conditions (50 °C, 3 bar, 0.4 L/kg) resulted in the maximum removal of CO_2_ of 180 g CO_2_/kg slag (Table 1). However, this still resulted in only a 50% conversion yield of calcium to carbonate [59]. The acetic acid was used to improve the leaching performance from blast furnace slag [60,61]. The addition of NaOH to increase the pH of the solution was required for the removal of CO_2_ at temperatures of 30–70 °C and at pressures of 1 or 30 bar. Therefore, about 4.4 kg of blast furnace slag, 3.6 L of acetic acid, and 3.5 kg of NaOH would be required to bind 1 kg of CO_2_. Moreover, the additional subproducts, in the form of the heat that would be necessary for acetic acid evaporation and the electricity that would be required for NaOH regeneration make the steel-making slag carbonation unreasonable [60]. 

Basic oxygen furnace slag (BOF) is among the steel-making slags (ultra-fine, fly ash, and blended hydraulic cement slags) with the highest CaO contents (35–56%) [11,62,63]. Moreover, the CaO in BOF slags is characterized by high reactivity, which means that its surface is readily soluble. The CaO soluble content in BOF slags (10%) is higher than in electrical arc furnace (EAF) slags (3%) [64]. The CO_2_ removal with BOF was measured at 63 g CO_2_/kg of BOF slag [65]. The enhancement of this value was obtained with optimized slurry preparation (L/S ratio of 0.05–0.2) and dynamic conditions in the field-scale upgrading column, which resulted in a CO_2_ removal of 73 g CO_2_/kg of BOF slag. The decisive parameter affecting the effectiveness of CO_2_ removal was the particle size of the BOF slag. Fine BOF slag (<0.106  mm) showed the maximum CO_2_ removal performance (300 g CO_2_/ kg BOF slag) (Table 1). This high removal capacity was confirmed by the 100% conversion of calcium to carbonate [66]. 

**Table 1:** A table summarizing the potential of waste/residue to remove CO_2_.

Waste/Residue	Calcium Content	Maximum CO_2_ Removal	Reference
Ash bottom	22–53%	23.5 mL/g	[42]
Palm oil ash	9.65%	53% reduction	[44]
Wood ash	24–46%	200 g/kg 115 g/kg	[50] [48]
Steel-making slag	15–42%	180 g/kg	[58]
Basic oxygen furnace slag	35–56%	300 g/kg	[65]
Air pollution control residues	38% Ca(OH) 28% CaClOH	-	[29]
Stabilized wastewater anaerobic sludge	35.1%	127.2 g/kg	[66]

### 5.3. Air Pollution Control Residues

Another type of industrial solid residue that could be used for CO_2_ sequestration is air pollution control (APC) residues, an alkaline residue that can be collected from various incinerator plant flue gas clean-up systems. The amount of calcium available for carbonation was estimated to be 38% in the form of Ca(OH)_2_ and 29% in the form of CaClOH (Table 1). The temperature required for the effective dry carbonation of APC residues is about 400 °C. However, the slurry carbonation of these residues might be lowered to 30 °C [29]. Currently, the APC residues are mostly tested in the regeneration step, which is described above. The carbonation also reduced the leaching of several elements from the APC residues. The carbonated APC residue leaching of Pb was below the limits set for nonhazardous waste landfills and those of Cu and Zn were below the limits set for inert waste landfills. However, the leaching of Cr was only slightly affected by carbonation, while the leaching of Sb increased after carbonation to the values dedicated to hazardous waste landfills. The concentration of chlorides in the eluate also still largely exceeded the values for hazardous waste [29]. 

### 5.4. Wastewater Anaerobic Sludge

The innovative solution for CO_2_ removal is wastewater anaerobic sludge stabilized with calcium oxide, which was prepared from 5 g of separated solids mixed with 1 g of CaO [67]. The content of calcium in the sludge increased to 351 mg/g. CO_2_ started to appear in the outlet stream after 250 min at a flow rate of 15 mL/min during biogas upgrading. The maximum CO_2_ removal level was 127.22 ± 1.5 mg CO_2_/g of stabilized sludge (Table 1). Biomethane concentration in the biogas increased from 56.5 ± 1.7% in the raw biogas to 98.9 ± 0.2% [67].

## 6. Types of Reactors Used for Biogas Upgrading

The most common reactor for CO_2_ removal is a column-packed bed reactor [42,67] or bubble column reactor [68]. It is recommended that before conducting experiments with this type of reactor, nitrogen should be introduced to purge the air/O_2_ from the reactor to avoid the possibility of creating explosive mixtures with CH_4_ within the reactor [69]. The bed inside the packed-bed type of reactor depends on the absorbent type. For solid absorbent materials such as ashes, this is located on a drilled plate, covered, for example, by a geotextile fabric that will retain the small particles and allow gas to flow evenly, avoiding the formation of preferential gas pathways [47]. The amount of ash and the thickness of the layers is defined by the specific flow rate and volumetric gas flow rate. It is recommended that researchers should avoid the excessive packing of the reactor and facilitate its filling and emptying. Inside the reactor, the empty volume below the ash layer ensures the proper distribution of the upgraded biogas, while the empty volume above the ash layer is used to collect the biomethane at the top of the reactor [47]. The bubble column reactor is a cylindrical column filled with liquid; at the bottom, it is equipped with a perforated plate gas distributor [69,70]. The biogas is upgraded by flowing through column and exits at the top bubble column. This type of reactor was also employed with packing material, which is commonly known by the trade name of “plastic bioball”, having an overall spherical shape and with uniform and structured spikes around the body and a high surface-area-to-volume ratio of 1895 m^2^/m^3^ [15]. This type of packing ensures good gas-liquid phase contact. Another type of reactor, a high-gravity rotating packed bed (HGRPB), was also constructed to enhance the contact and mass transfer between the phases. The flow of gas/liquid into the reactor forms a centrifugal eddy current between the phases, greatly enhancing the mass transfer between the gas and liquid phases. The phases making contact in the reactor create thinner film membranes (1–10 μm) or smaller droplets (10–100 μm) that increase the contact surface. The raw biogas is introduced from the bottom of the reactor and liquid is evenly sprayed inside using centrifugal force via a mesh distribution system. After upgrading, the enriched biogas flows from the top of the reactor and the liquid is removed below the bed [71]. Another solution that enhances the contact gas–liquid is the T-shaped microchannel [72]. This device was proposed to purify biogas with seawater containing 0.1 wt % of Iranian-modified clinoptilolite zeolite and several precipitates (i.e., water distillation, phosphogypsum, and a power plant clarifier unit). However, the authors suggest that it is possible to use other forms of industrial waste containing CaO where CaCO_3_ precipitate can be formed. A T-typed microchannel has a channel length of 25 cm and a circular cross-section, with an internal diameter of 800 μm. Carbon dioxide molecules are absorbed by passing through a two-phase medium and by strongly mixing the biogas and liquid flows entering the micromixer. The liquid is then pumped into the microchannel with a syringe pump. The microchannel outflow separates into the gas and liquid phases, while passing a Büchner flask as a flash drum. Finally, the recovered biogas loses its residual moisture after passing through a water trap.

## 7. Conclusions and Perspectives on Alternative Upgrading Biogas Technologies Using Wastes

The new technologies using wastes for upgrading biogas during mineral carbonation are promising. The most important criterion is that they allow for saving more CO_2_ than the commercially available technologies. However, during consideration of the most appropriate technology, economic reasons should be taken into account, such as:

- the amount of waste that should be delivered for the biogas upgrading facility;
- the distance between the upgrading facility and the facility delivering the waste;
- the costs of treatment of the wastewater that is eventually produced;
- the possibility of the regeneration of the reagents used in the process;
- the characteristic of the carbonated waste and the possibility of its application.

## Data Availability

Not applicable.

## References

[1] Rasi S., Veijanen A., Rintala J. (2007). Trace compounds of biogas from different biogasproduction plants. Energy.

[2] Goswami R., Chattopadhyay P., Shome A., Banerjee S.N., Chakraborty A.K., Mathew A.K., Chaudhury S. (2016). An Overview of Physico-Chemical Mechanisms of Biogas Production by Microbial Communities: A Step towards Sustainable Waste Management. 3 Biotech.

[3] Soreanu G., Béland M., Falletta P., Edmonson K., Svoboda L., Al-Jamal M., Seto P. (2011). Approaches concerning siloxane removal from biogas—A review. Can. Biosyst. Eng..

[4] Persson M., Jönsson O., Wellinger A. (2006). Biogas Upgrading to Vehicle Fuel Standards and Grid Injection. International Energy Agency IEA Bioenergy. http://www.iea-biogas.net/_download/publi-task37/upgrading_report_final.pdf.

[5] Nizami A.S., Murphy J.D. (2010). What type of digester configurations should be employed to produce biomethane from grass silage?. Renew. Sustain. Energy Rev..

[6] Zhao Q., Leonhardt E., MacConnell C., Frear C., Chen S. (2010). Purification technologies for biogas generated by anaerobic digestion. Climate friendly farming, compressed biomethane. CSANR Research Report 2010-001 (Chapter 9).

[7] Karne H., Mahajan U., Ketkar U., Kohade A., Khadilkar P., Mishra A. (2023). A Review on Biogas Upgradation Systems. Mater. Today Proc..

[8] Feroskhan M., Ismail S. (2017). A Review on the Purification and Use of Biogas in Compression Ignition Engines. Int. J. Automot. Mech. Eng..

[9] Adnan A.I., Yin Ong M., Nomanbhay S., Chew K.W., Show P.L. (2019). Technologies for Biogas Upgrading to Biomethane: A Review. Bioengineering.

[10] Muñoz R., Meier L., Diaz I., Jeison D. (2015). A Review on the State-of-the-Art of Physical/Chemical and Biological Technologies for Biogas Upgrading. Rev. Environ. Sci. Biotechnol..

[11] Sanna A., Uibu M., Caramanna G., Kuusik R., Maroto-Valer M.M. (2014). A Review of Mineral Carbonation Technologies to Sequester CO_2_. Chem. Soc. Rev..

[12] Wilcox J., Baciocchi R., Costa G., Polettini A., Pomi R., Stramazzo A., Zingaretti D. (2016). Accelerated Carbonation of Steel Slags Using CO_2_ Diluted Sources: CO_2_ Uptakes and Energy Requirements. Front. Energy Res..

[13] Fei Z., Bao Q., Zheng X., Zhang L., Wang X., Wei Y., Yan S., Ji L. (2022). Glycinate-Looping Process for Efficient Biogas Upgrading and Phytotoxicity Reduction of Alkaline Ashes. J. Clean. Prod..

[14] Ji L., Zhang L., Zheng X., Feng L., He Q., Wei Y., Yan S. (2021). Simultaneous CO_2_ Absorption, Mineralisation and Carbonate Crystallisation Promoted by Amines in a Single Process. J. CO2 Util..

[15] Tippayawong N., Thanompongchart P. (2010). Biogas Quality Upgrade by Simultaneous Removal of CO_2_ and H_2_S in a Packed Column Reactor. Energy.

[16] Mamun M.R., Karim M.R., Rahman M.M., Asiri A.M., Torii S. (2016). Methane Enrichment of Biogas by Carbon Dioxide Fixation with Calcium Hydroxide and Activated Carbon. J. Taiwan Inst. Chem. Eng..

[17] Katariya H.G., Patolia H.P. (2021). Methane Enrichment in Biogas by Using Aqueous Solutions of Alkaline Salts. Biomass Convers. Biorefin..

[18] Chinea L., Slopiecka K., Bartocci P., Alissa Park A.H., Wang S., Jiang D., Fantozzi F. (2023). Methane Enrichment of Biogas Using Carbon Capture Materials. Fuel.

[19] Rattanaya T., Manmeen A., Kongjan P., Bunyakan C., Reungsang A., Prasertsit K., Lombardi L., Jariyaboon R. (2021). Upgrading Biogas to Biomethane Using Untreated Groundwater-NaOH Absorbent: Pilot-Scale Experiment and Scale-up Estimation for a Palm Oil Mill. J. Water Process Eng..

[20] Maile O.I., Tesfagiorgis H., Muzenda E., Filho L.W., Surroop D. (2017). Possible Absorbent Regeneration in Biogas Purification and Upgrading: A Review. The Nexus: Energy, Environment and Climate Change. Green Energy and Technology.

[21] Zhang W., Liu H., Sun Y., Cakstins J., Sun C., Snape C.E. (2016). Parametric Study on the Regeneration Heat Requirement of an Amine-Based Solid Adsorbent Process for Post-Combustion Carbon Capture. Appl. Energy.

[22] Zhang M., Guo Y. (2014). A Comprehensive Model for Regeneration Process of CO_2_ Capture Using Aqueous Ammonia Solution. Int. J. Greenh. Gas Control.

[23] Leonzio G. (2016). Recovery of Metal Sulphates and Hydrochloric Acid from Spent Pickling Liquors. J. Clean. Prod..

[24] Librandi P., Costa G., de Souza A.C.B., Stendardo S., Luna A.S., Baciocchi R. (2017). Carbonation of Steel Slag: Testing of the Wet Route in a Pilot-Scale Reactor. Energy Procedia.

[25] Said A., Mattila H.P., Järvinen M., Zevenhoven R. (2013). Production of Precipitated Calcium Carbonate (PCC) from Steelmaking Slag for Fixation of CO_2_. Appl. Energy.

[26] Baciocchi R., Costa G., Gavasci R., Lombardi L., Zingaretti D. (2012). Regeneration of a Spent Alkaline Solution from a Biogas Upgrading Unit by Carbonation of APC Residues. Chem. Eng. J..

[27] Baciocchi R., Carnevale E., Costa G., Lombardi L., Olivieri T., Paradisi A., Zanchi L., Zingaretti D. (2013). Pilot-Scale Investigation of an Innovative Process for Biogas Upgrading with CO_2_ Capture and Storage. Energy Procedia.

[28] Wang Y., Liu J., Hu X., Chang J., Zhang T., Shi C. (2022). Utilization of accelerated carbonation to enhance the application of steel slag: A review. J. Sustain. Cement-Based Mater..

[29] Baciocchi R., Costa G., Polettini A., Pomi R., Prigiobbe V. (2009). Comparison of Different Reaction Routes for Carbonation of APC Residues. Energy Procedia.

[30] Lombardi L., Carnevale E. (2013). Economic Evaluations of an Innovative Biogas Upgrading Method with CO_2_ Storage. Energy.

[31] Baena-Moreno F.M., Rodríguez-Galán M., Reina T.R., Zhang Z., Vilches L.F., Navarrete B. (2019). Understanding the Effect of Ca and Mg Ions from Wastes in the Solvent Regeneration Stage of a Biogas Upgrading Unit. Sci. Total Environ..

[32] Baena-Moreno F.M., Reina T.R., Rodríguez-Galán M., Navarrete B., Vilches L.F. (2021). Synergizing Carbon Capture and Utilization in a Biogas Upgrading Plant Based on Calcium Chloride: Scaling-up and Profitability Analysis. Sci. Total Environ..

[33] Baena-Moreno F.M., Rodríguez-Galán M., Vega F., Reina T.R., Vilches L.F., Navarrete B. (2019). Synergizing Carbon Capture Storage and Utilization in a Biogas Upgrading Lab-Scale Plant Based on Calcium Chloride: Influence of Precipitation Parameters. Sci. Total Environ..

[34] Arti M., Youn M.H., Park K.T., Kim H.J., Kim Y.E., Jeong S.K. (2017). Single process for CO_2_ capture and mineralization in various alkanolamines using calcium chloride. Energy Fuels.

[35] Galvez-Martos J.L., Elhoweris A., Morrison J., Al-Horr Y. (2018). Conceptual design of a CO_2_ capture and utilisation process based on calcium and magnesium rich brines. J. CO2 Util..

[36] Erdogan N., Eken H.A. (2017). Precipitated calcium carbonate production, synthesis and properties. Phys. Probl. Min. Process.

[37] Dong C., Song X., Li Y., Liu C., Chen H., Yu J. (2018). Impurity ions effect on CO2 mineralization via coupled reaction-extraction-crystallization process of CaCl2 waste liquids. J. CO2 Util..

[38] Wiles C.C. (1996). Municipal Solid Waste Combustion Ash: State-of-the-Knowledge. J. Hazard. Mater..

[39] Costa G., Baciocchi R., Polettini A., Pomi R., Hills C.D., Carey P.J. (2007). Current status and perspectives of accelerated carbonation processes on municipal waste combustion residues. Environ. Monit. Assess.

[40] Mostbauer P., Lenz S.S., Lechner P. (2008). MSWI bottom ash for upgrading of biogas and landfill gas. Environ. Technol..

[41] del Valle-Zermeño R., Romero-Güiza M.S., Chimenos J.M., Formosa J., Mata-Alvarez J., Astals S. (2015). Biogas Upgrading Using MSWI Bottom Ash: An Integrated Municipal Solid Waste Management. Renew. Energy.

[42] Yao Z., Prabhakar A.K., Cadiam Mohan B., Wang C.H. (2022). An Innovative Accelerated Carbonation Process for Treatment of Incineration Bottom Ash and Biogas Upgrading. Waste Manag..

[43] Rendek E., Ducom G., Germain P. (2007). Influence of Waste Input and Combustion Technology on MSWI Bottom Ash Quality. Waste Manag..

[44] Rattanaya T., Kongjan P., Cheewasedtham C., Bunyakan C., Yuso P., Cheirsilp B., Jariyaboon R. (2022). Application of Palm Oil Mill Waste to Enhance Biogas Upgrading and Hornwort Cultivation. J. Environ. Manag..

[45] Foo K.Y., Hameed B.H. (2009). Value-Added Utilization of Oil Palm Ash: A Superior Recycling of the Industrial Agricultural Waste. J. Hazard. Mater..

[46] Chavez R.-H., Guadarrama J.J. (2015). Biogas Treatment by Ashes from Incineration Processes. Clean Technol. Environ. Policy.

[47] Lombardi L., Costa G., Spagnuolo R. (2018). Accelerated Carbonation of Wood Combustion Ash for CO_2_ Removal from Gaseous Streams and Storage in Solid Form. Environ. Sci. Pollut. Res..

[48] Papurello D., Silvestri S., Biasioli F., Lombardi L. (2022). Wood Ash Biomethane Upgrading System: A Case Study. Renew. Energy.

[49] Vassilev S.V., Vassileva C.G., Petrova N.L. (2021). Mineral Carbonation of Biomass Ashes in Relation to Their CO_2_ Capture and Storage Potential. ACS Omega.

[50] Andersson J., Nordberg A. (2017). Biogas Upgrading Using Ash from Combustion of Wood Fuels: Laboratory Experiments. Energy Environ. Res..

[51] Mulu E., M’Arimi M.M., Ramkat R.C., Mecha A.C. (2021). Potential of wood ash in purification of biogas. Energy Sustain. Dev..

[52] Wang L., Jin Y., Nie Y. (2010). Investigation of accelerated and natural carbonation of MSWI fly ash with a high content of Ca. J. Hazard. Mater..

[53] Koch R., Sailer G., Paczkowski S., Pelz S., Poetsch J., Müller J., Frusteri F. (2021). Lab-Scale Carbonation of Wood Ash for CO_2_-Sequestration. Energies.

[54] Doucet F.J. (2010). Effective CO_2_-Specific Sequestration Capacity of Steel Slags and Variability in Their Leaching Behavior in View of Industrial Mineral Carbonation. Min. Eng..

[55] Chen B., Yoon S., Zhang Y., Han L., Choi Y. (2019). Reduction of Steel Slag Leachate PH via Humidification Using Water and Aqueous Reagents. Sci. Total Environ..

[56] Heiderscheidt E., Postila H., Leiviskä T. (2020). Removal of Metals from Wastewaters by Mineral and Biomass-Based Sorbents Applied in Continuous-Flow Continuous Stirred Tank Reactors Followed by Sedimentation. Sci. Total Environ..

[57] Stolaroff J.K., Lowry G.V., Keith D.W. (2005). Using CaO- and MgO-Rich Industrial Waste Streams for Carbon Sequestration. Energy Convers. Manag..

[58] Truong M.V., Nguyen L.N., Li K., Fu Q., Johir M.A.H., Fontana A., Nghiem L.D. (2020). Biomethane Production from Anaerobic Co-Digestion and Steel-Making Slag: A New Waste-to-Resource Pathway. Sci. Total Environ..

[59] Baciocchi R., Costa G., Di Bartolomeo E., Polettini A., Pomi R. (2011). Wet versus slurry carbonation of EAF steel slag. Greenh. Gas Sci. Technol..

[60] Eloneva S., Teir S., Salminen J., Fogelholm C.J., Zevenhoven R. (2008). Fixation of CO_2_ by Carbonating Calcium Derived from Blast Furnace Slag. Energy.

[61] Teir S., Eloneva S., Fogelholm C.J., Zevenhoven R. (2007). Dissolution of Steelmaking Slags in Acetic Acid for Precipitated Calcium Carbonate Production. Energy.

[62] Chang E.E., Chen C.H., Chen Y.H., Pan S.Y., Chiang P.C. (2011). Performance Evaluation for Carbonation of Steel-Making Slags in a Slurry Reactor. J. Hazard. Mater..

[63] Proctor D.M., Fehling K.A., Shay E.C., Wittenborn J.L., Green J.J., Avent C., Bigham R.D., Connolly M., Lee B., Shepker T.O. (2000). Physical and Chemical Characteristics of Blast Furnace, Basic Oxygen Furnace, and Electric Arc Furnace Steel Industry Slags. Environ. Sci. Technol..

[64] Motz H., Geiseler J. (2001). Products of Steel Slags an Opportunity to Save Natural Resources. Waste Manag..

[65] Sarperi L., Surbrenat A., Kerihuel A., Chazarenc F. (2014). The Use of an Industrial By-Product as a Sorbent to Remove CO_2_ and H_2_S from Biogas. J. Environ. Chem. Eng..

[66] Chetri J.K., Reddy K.R., Grubb D.G. (2020). Carbon-Dioxide and Hydrogen-Sulfide Removal from Simulated Landfill Gas Using Steel Slag. J. Environ. Eng..

[67] Zieliński M., Karczmarczyk A., Kisielewska M., Dębowski M. (2022). Possibilities of Biogas Upgrading on a Bio-Waste Sorbent Derived from Anaerobic Sewage Sludge. Energies.

[68] Mostbauer P., Lombardi L., Olivieri T., Lenz S. (2014). Pilot Scale Evaluation of the BABIU Process—Upgrading of Landfill Gas or Biogas with the Use of MSWI Bottom Ash. Waste Manag..

[69] Madhania S., Kusdianto K., Machmudah S., Nurtono T., Widiyastuti W., Winardi S. (2020). Biogas quality upgrading by carbon mineralization with calcium hydroxide solution in continuous bubble column reactor. AIP Conf. Proc..

[70] Wang W., Hu M., Zheng Y., Wang P., Ma C. (2011). CO_2_ fixation in Ca_2_/Mg-rich aqueous solutions through enhanced carbonate precipitation. Ind. Eng. Chem. Res..

[71] Tran L., Le T., Nguyen T., Tran Q., Le X., Pham M., Lam V., Van Do M. (2021). Simultaneous removal efficiency of H_2_S and CO_2_ by high-gravity rotating packed bed: Experiments and simulation. Open Chem..

[72] Aghel B., Gouran A., Behaien S., Vaferi B. (2022). Experimental and Modeling Analyzing the Biogas Upgrading in the Microchannel: Carbon Dioxide Capture by Seawater Enriched with Low-Cost Waste Materials. Environ. Technol. Innov..

